# Supplementary material for: Neurotransmitter recognition by human vesicular monoamine transporter 2
Source: Nat Commun. 2024 Sep 16;15:7661. doi: 10.1038/s41467-024-51960-z (PMC11405867; doi:10.1038/s41467-024-51960-z)
Supplement: Supplementary file 1 — Supplementary Information [file 41467_2024_51960_MOESM1_ESM.pdf]

Supplementary Information

Neurotransmitter Recognition by Human Vesicular Monoamine Transporter 2

D. Im *et al.*

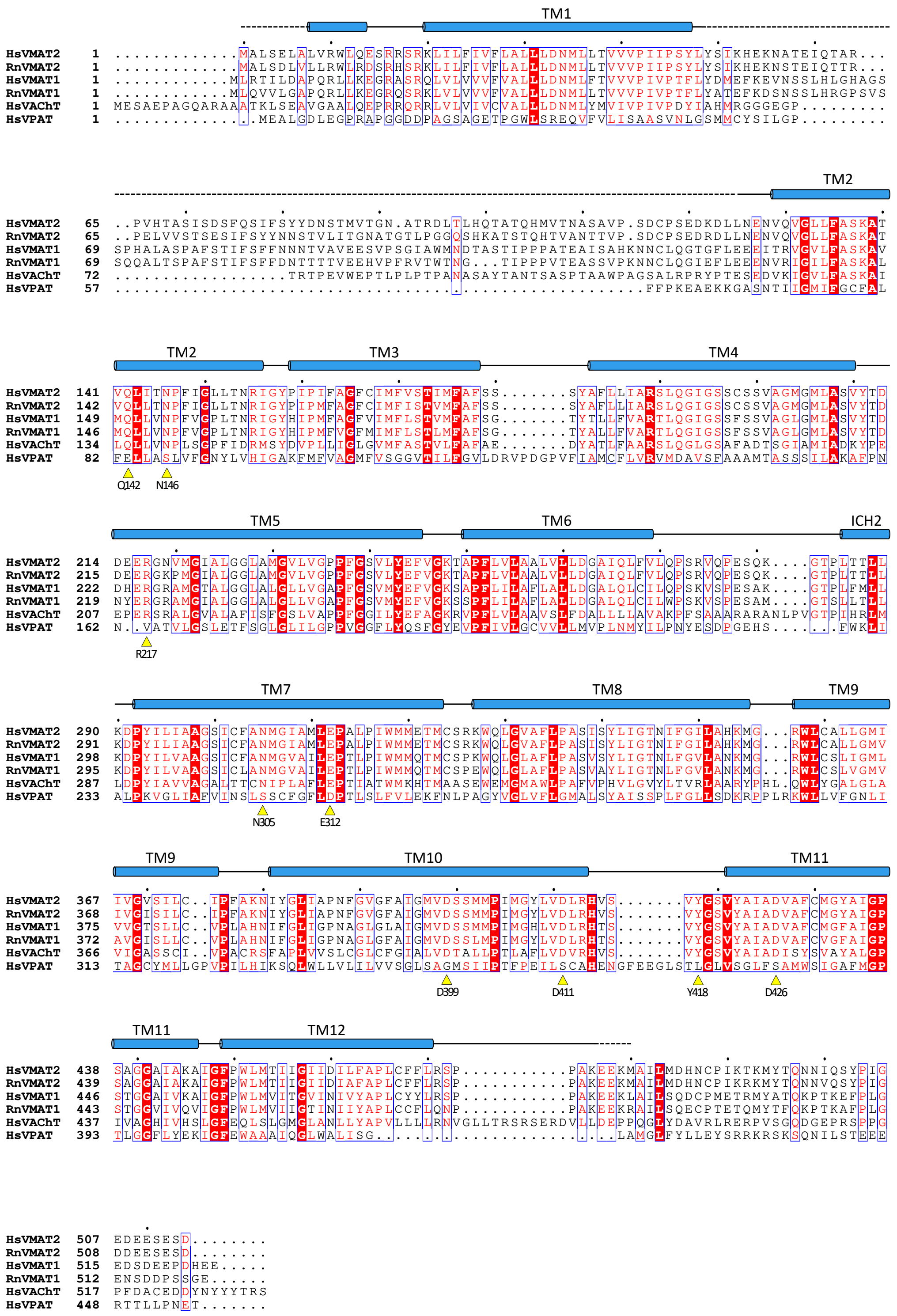

Supplementary Fig. 1 | Multiple sequence alignment of SLC18 family proteins. The sequences of the *Homo sapiens* VMAT2 (HsVMAT2), *Rattus norvegicus* VMAT2 (RnVMAT2), *Homo sapiens* VMAT1 (HsVMAT1), *Rattus norvegicus* VMAT1 (RnVMAT1), *Homo sapiens* VAChT

(HsVAChT) and *Homo sapiens* VPAT (HsVPAT) are aligned using CLUSTALW (https://www.genome.jp/tools-bin/clustalw). The secondary structure information for VMAT2 used in this structural analysis is shown at the top of the sequence. α helices are shown as cylinders. Unmodeled residues are shown as dashed lines. Conserved residues are colored as red and blue boxes. The key residues discussed throughout the article are indicated by yellow triangles.

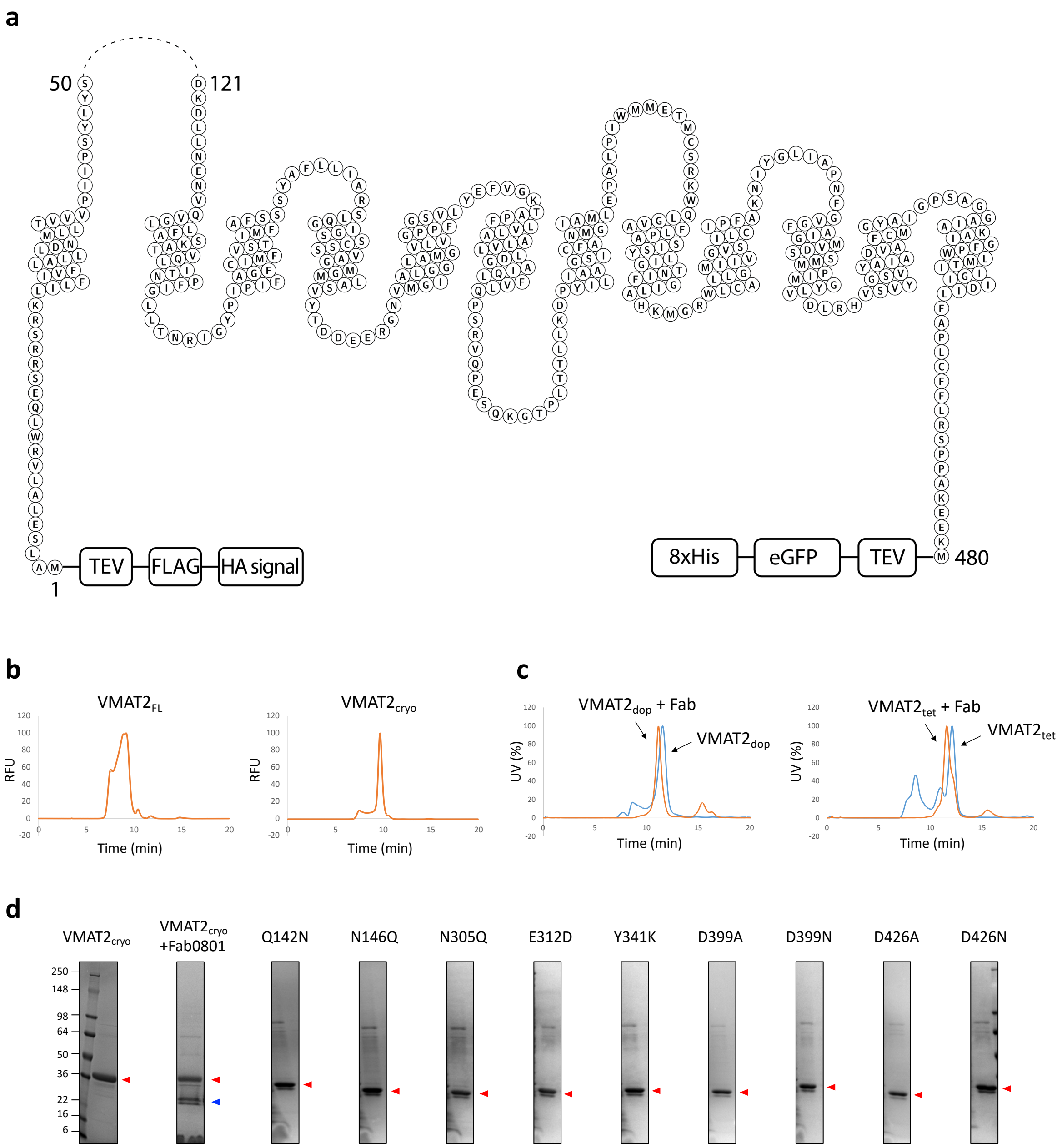

Supplementary Fig. 2 | Sample preparation of VMAT2-Fab0801 complex. a, Snake-shape diagram of the VMAT2 construct used in complex assembling. The snake-diagram of VMAT2 was adopted and modified from the Protter website (https://wlab.ethz.ch/protter/). **b**, Fluorescence size exclusion chromatography profiles of wild-type VMAT2 ($VMAT2_{FL}$) and VMAT2 construct used for cryo-EM ($VMAT2_{cryo}$). **c**, Size exclusion chromatography (SEC) profiles of $VMAT2_{cryo}$ and $VMAT2_{cryo}$-Fab0801 complex. **d**, SDS-PAGE results of $VMAT2_{cryo}$, $VMAT2_{cryo}$-Fab0801

complex, and each mutant VMAT2 after SEC. Red and blue arrows indicate VMAT2 and Fab regions, respectively.

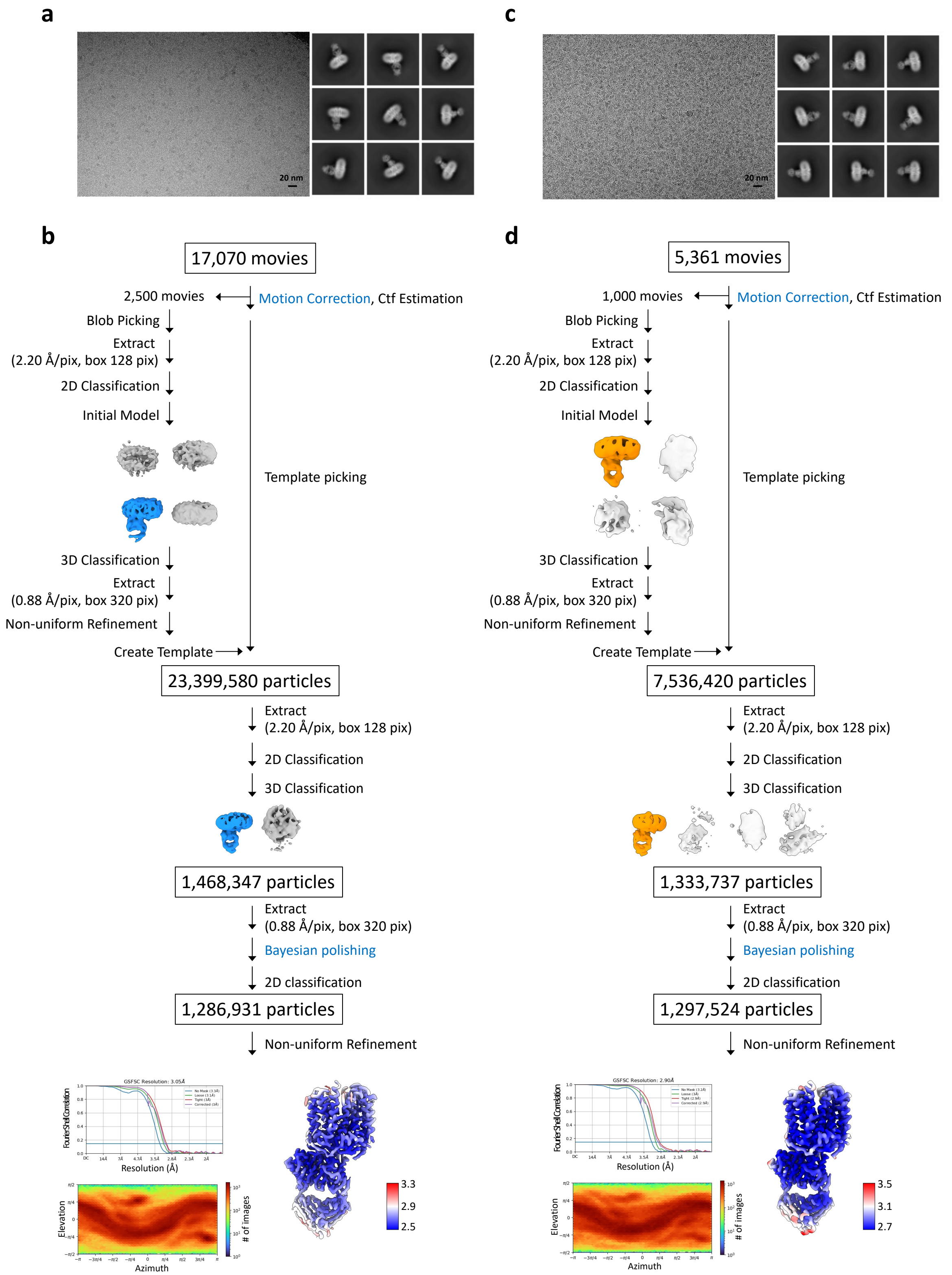

a
20 nm
c
20 nm
b
17,070 movies
2,500 movies
Motion Correction, Ctf Estimation
Blob Picking
Extract
(2.20 Å/pix, box 128 pix)
2D Classification
Initial Model
Template picking
3D Classification
Extract
(0.88 Å/pix, box 320 pix)
Non-uniform Refinement
Create Template
23,399,580 particles
Extract
(2.20 Å/pix, box 128 pix)
2D Classification
3D Classification
1,468,347 particles
Extract
(0.88 Å/pix, box 320 pix)
Bayesian polishing
2D classification
1,286,931 particles
Non-uniform Refinement
Resolution (Å)
Elevation
Azimuth
# of images
3.3
2.9
2.5
d
5,361 movies
1,000 movies
Motion Correction, Ctf Estimation
Blob Picking
Extract
(2.20 Å/pix, box 128 pix)
2D Classification
Initial Model
Template picking
3D Classification
Extract
(0.88 Å/pix, box 320 pix)
Non-uniform Refinement
Create Template
7,536,420 particles
Extract
(2.20 Å/pix, box 128 pix)
2D Classification
3D Classification
1,333,737 particles
Extract
(0.88 Å/pix, box 320 pix)
Bayesian polishing
2D classification
1,297,524 particles
Non-uniform Refinement
Resolution (Å)
Elevation
Azimuth
# of images
3.5
3.1
2.7

e

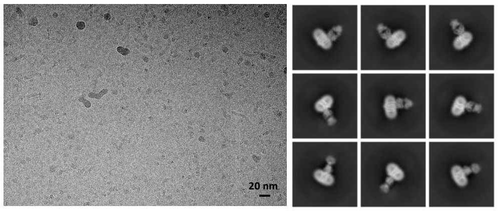

f

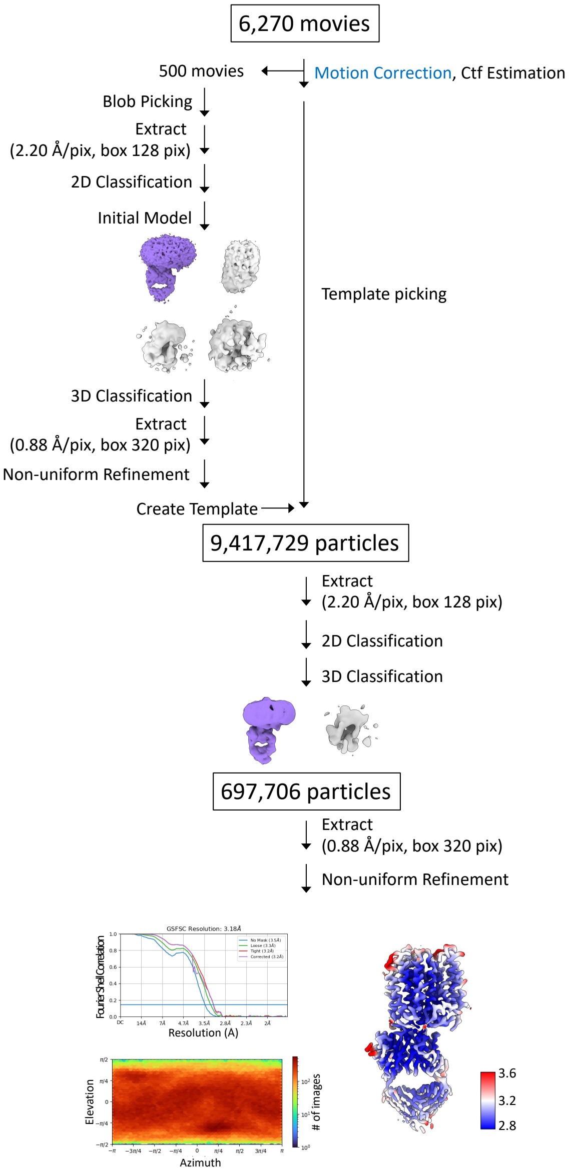

Supplementary Fig. 3 | Single-particle cryo-EM analysis procedure. a-f, Flow chart outlining cryo-EM image acquisition and processing performed to obtain structures of VMAT2. Representative cryo-EM micrographs and two-dimensional classification averages of VMAT2$_{apo}$

(**a**), VMAT2$_{dop}$ (**c**) and VMAT2$_{tet}$ (**e**). Cryo-EM data processing workflows of the VMAT2$_{apo}$ (**b**), VMAT2$_{dop}$ (**d**) and VMAT2$_{tet}$ (**f**). Data processing was performed by Relion (in blue) and cryoSPARC (in black).

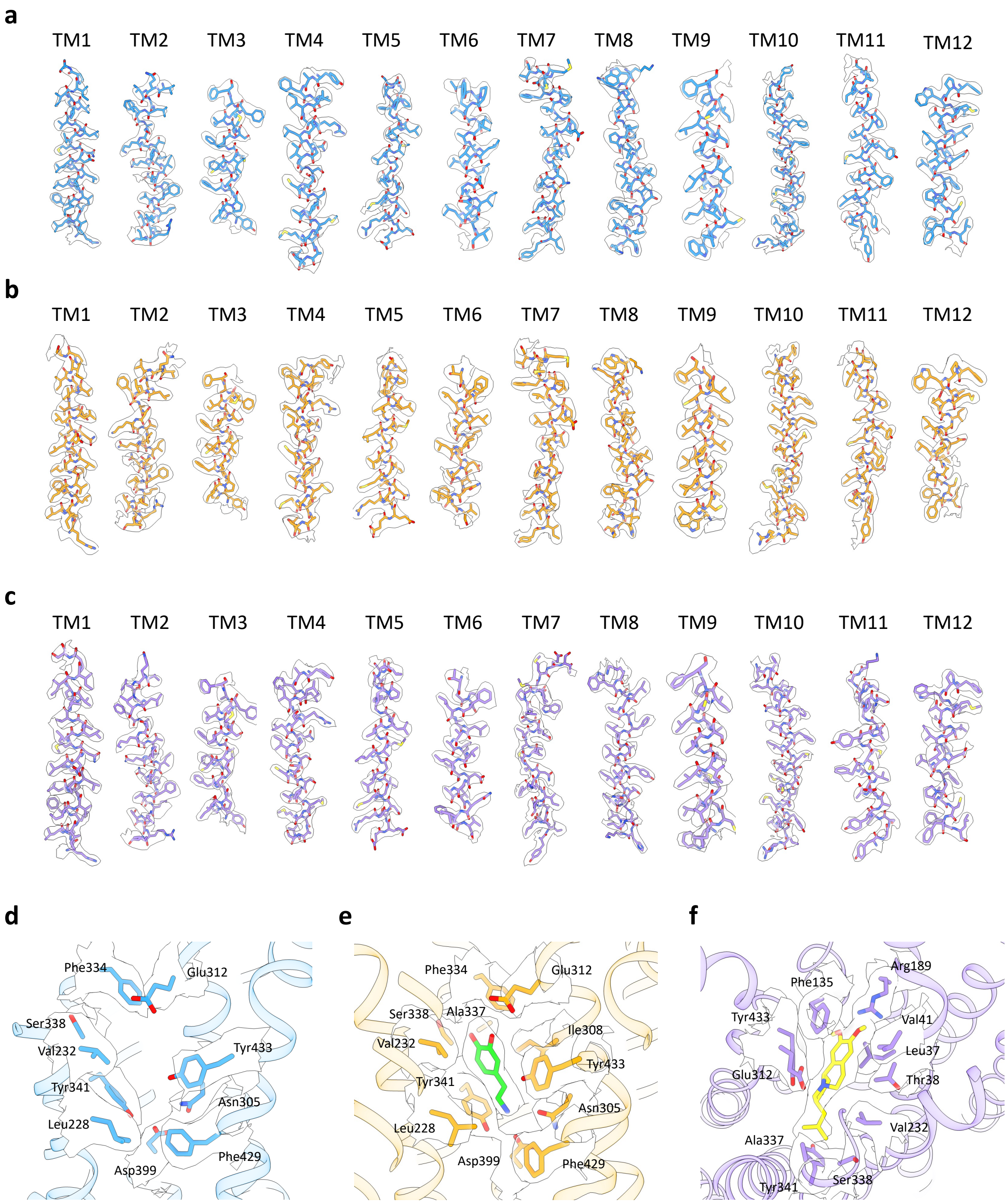

Supplementary Fig. 4 | Cryo-EM density map. a-c, The transmembrane helices 1-12 of the $VMAT2_{apo}$ (**a**), $VMAT2_{dop}$ (**b**) and $VMAT2_{tet}$ (**c**). **d**, The region of the $VMAT2_{apo}$ corresponding to the dopamine binding site. **c**, **d**, The ligand-binding site of $VMAT2_{dop}$ (**e**) and $VMAT2_{tet}$ (**f**).

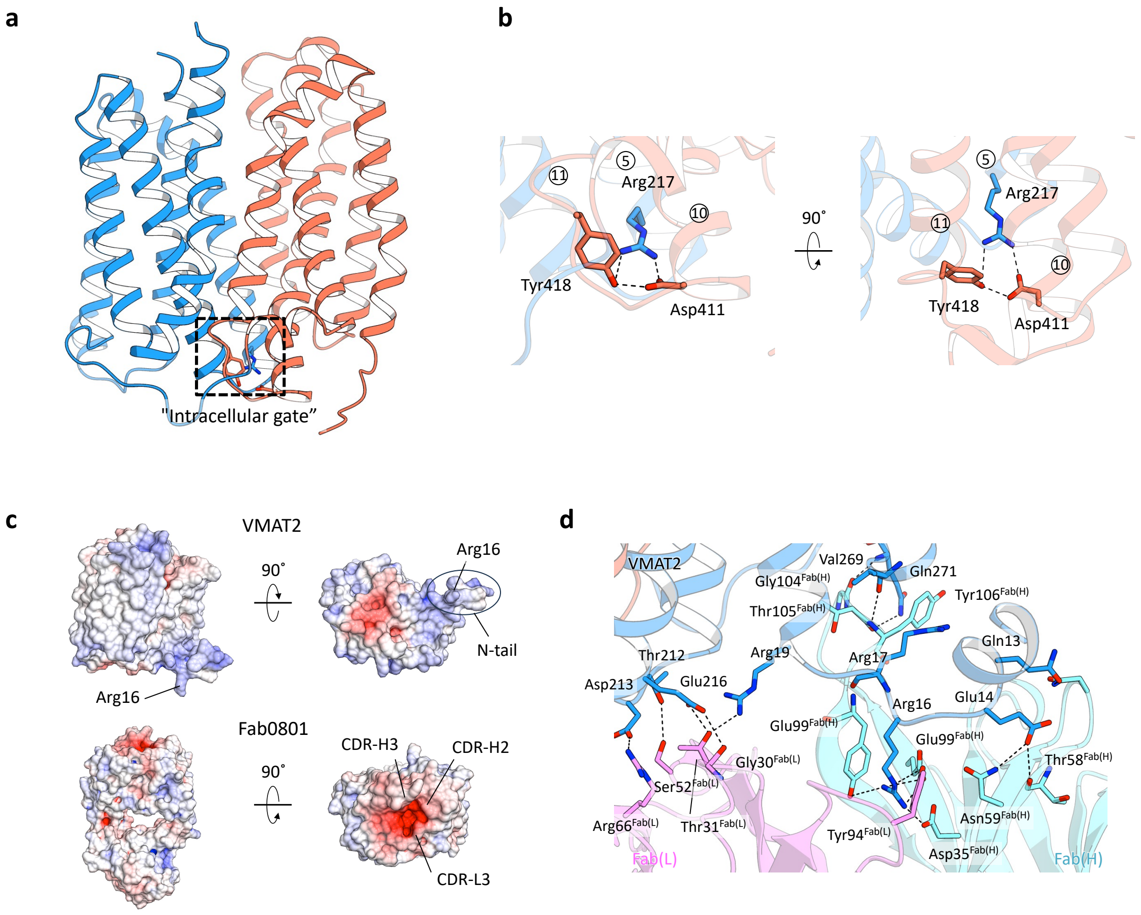

Supplementary Fig. 5 | Intracellular gate and intracellular-bound antibody of VMAT2. a, Side view of the VMAT2$_{apo}$. The dash square represents the intracellular gate. **b**, Close-up view of a dash square in (**a**). The triad of Arg217-Asp411-Tyr418 hydrogen bonded network. The N-terminal and C-terminal domain are indicated by blue and red. Proteins and residues are shown as ribbons and sticks. Dotted lines indicated hydrogen bonds. **c**, The electrostatic potential surface of VMAT2 and Fab0801. The positively charged surface of N-terminal tail of VMAT2 and the negatively charged surface of the CDRs of Fab0801 contribute to the complex formation. **d**, Close-up view of the complex interfaces between VMAT2 and Fab0801. The interfaces were stabilized by hydrogen bonds (dashed lines) and hydrophobic interactions between several key residues (sticks).

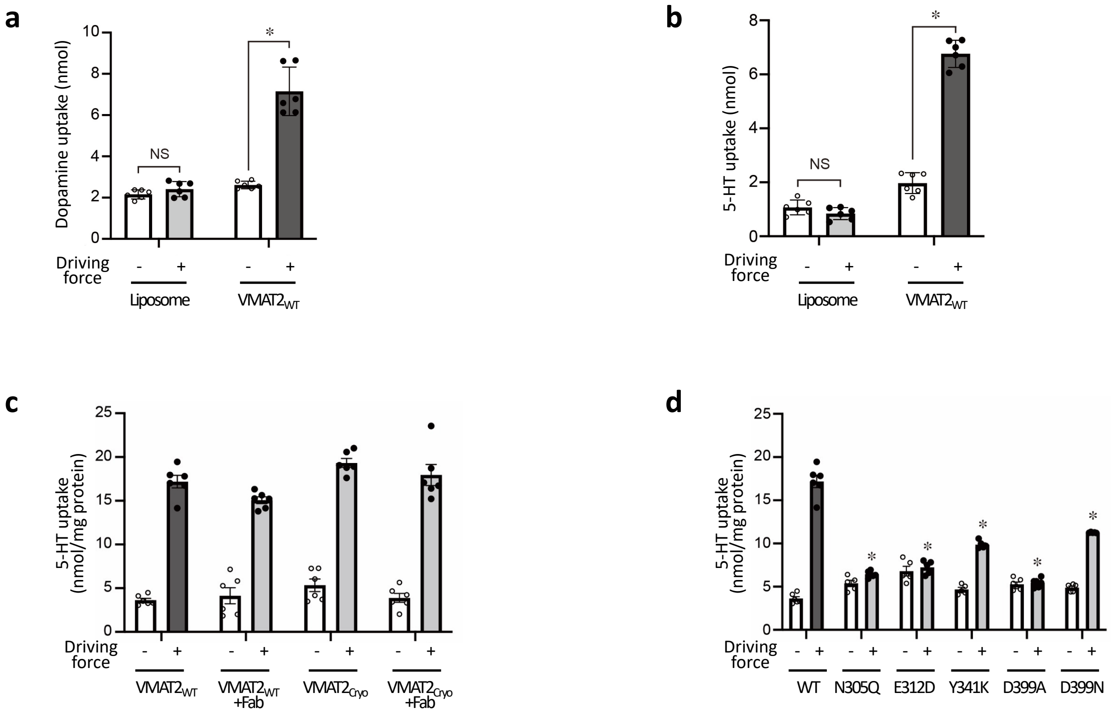

Supplementary Fig. 6 | Neurotransmitter transport activity of VMAT2 and its variants. **a**, **b**, Transport activity of dopamine (**a**) and 5-HT (**b**) in wild-type VMAT2 (VMAT2$_{WT}$) proteoliposomes. Error bars represent mean ± SEM; points represent biologically independent measurements (n = 6). *P < 0.01; NS, not significant. **c**, 5-HT transport activity of VMAT2$_{WT}$ and cryo-EM construct (VMAT2$_{Cryo}$) in the presence or absence of the intracellular recognition antibody Fab0801. **d**, Uptake of 5-HT by proteoliposomes containing purified VMAT2 variants. Error bars represent mean ± SEM; points represent biologically independent measurements (n = 5 for N305Q, E312D and D399N; n = 6 for others).

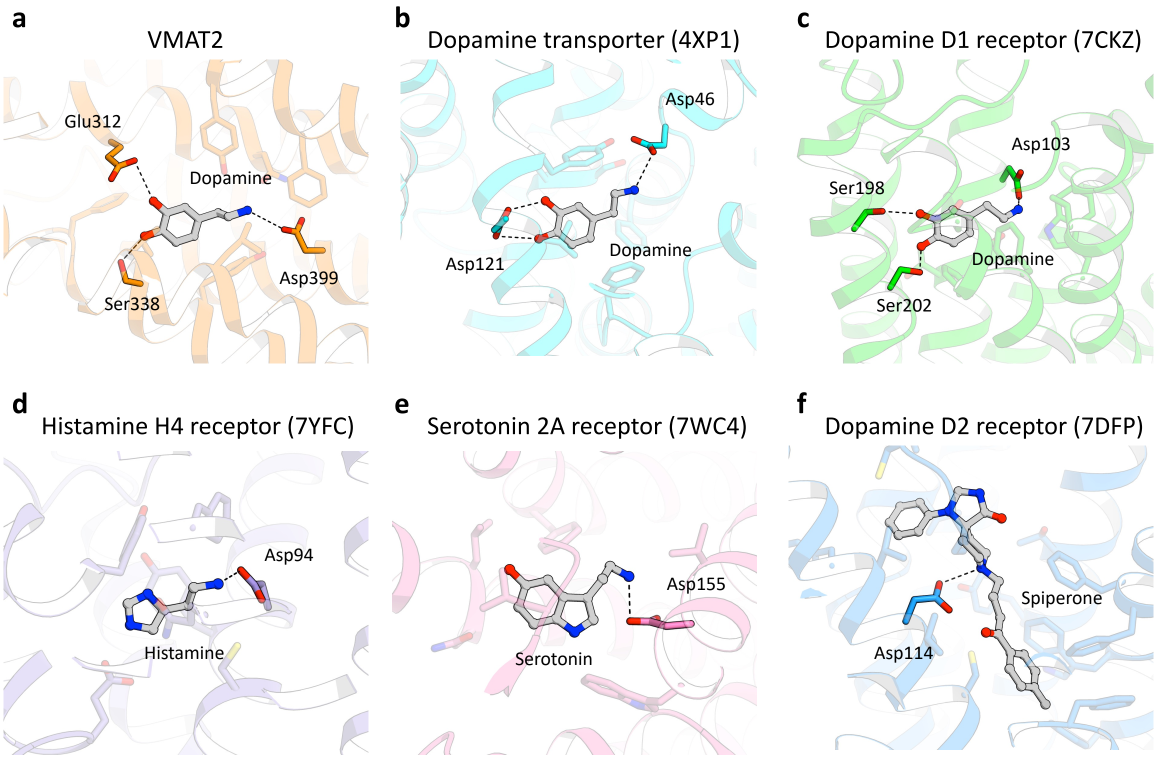

Supplementary Fig. 7 | Comparison of amine ligand binding features of transporters and G protein-coupled receptors (GPCRs). a-f, Proteins and ligands are shown as ribbons and sticks. Side chains are indicated by sticks. The substrate binding site on VMAT2 (**a**; orange), dopamine transporter (**b**; cyan, PDB 4XP1)[24], dopamine D1 receptor (**c**; green, PDB 7CKZ)[26], histamine H4 receptor (**d**; purple, PDB 7YFC)[28], 5-HT 2A receptor (**e**; pink, PDB 7WC4)[27] and dopamine D2 receptor (**f**; blue, PDB 7DFP)[25].

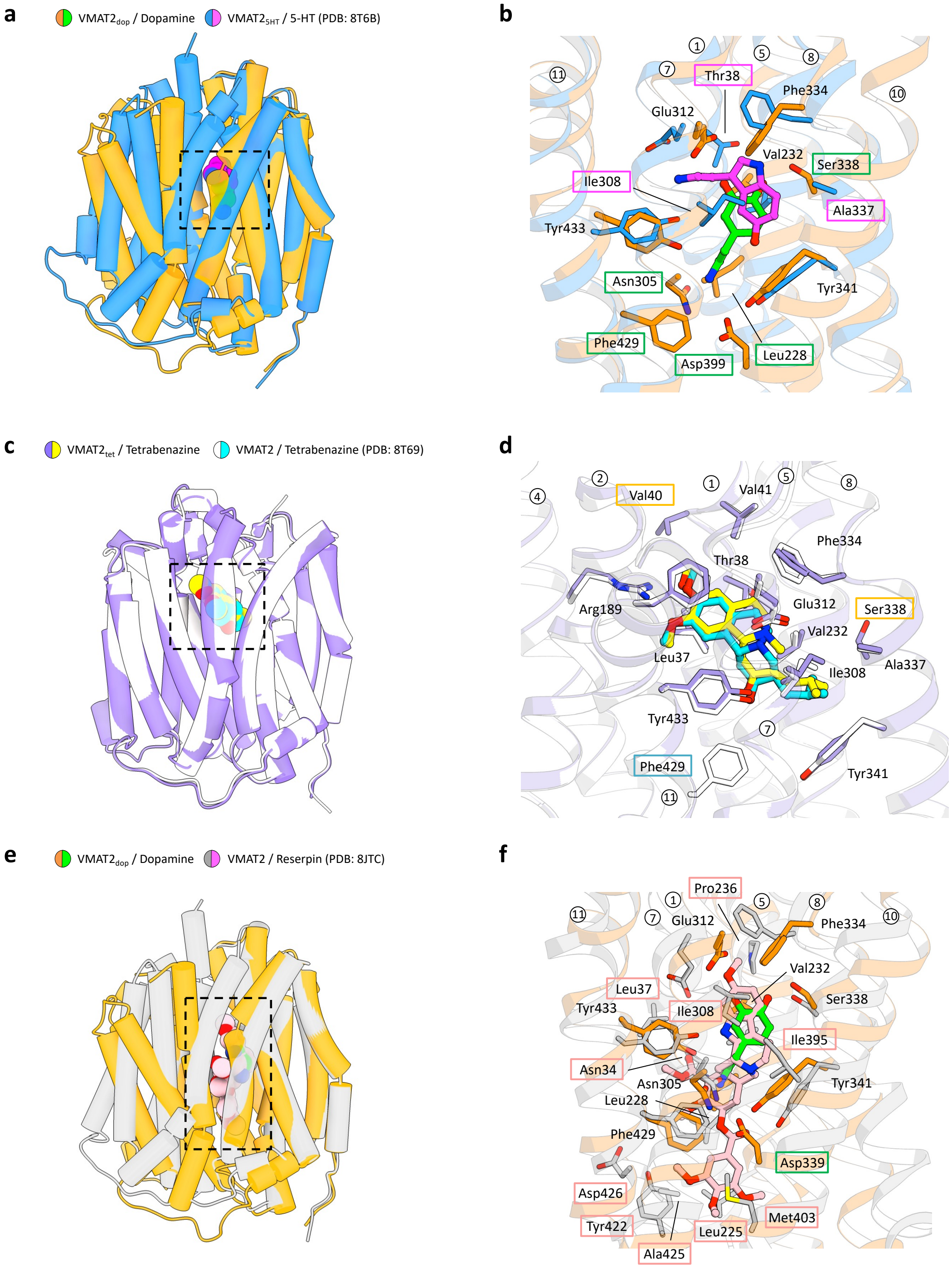

Supplementary Fig. 8 | Different ligand binding modes in VMAT2. **a**, Side view of the superposition of $VMAT2_{dop}$ (orange) and $VMAT2_{5HT}$ (blue, PDB 8T6B)[17]. **b**, Close-up view of a dash square in (**a**). Dopamine and 5-HT are indicated by green and magenta sticks, respectively. **c**, Side view of the superposition of $VMAT2_{tet}$ (purple) and tetrabenazine-bound VMAT2 (white, PDB 8T69)[17]. **d**, Close-up view of a dash square in (**c**). Tetrabenazine in each structure was

indicated by yellow ($VMAT2_{tet}$) and cyan (PDB 8T69) sticks, respectively. **e**, Side view of the superposition of $VMAT2_{dop}$ (orange) and reserpine-bound VMAT2 (gray, PDB 8T6A)[17]. **f**, Close-up view of a dash square in (**e**). Dopamine and reserpine are indicated by green and pink sticks, respectively. Proteins and ligands are shown as cylinders and CPK models (**a**, **c**, **e**). Residues that recognize each ligand alone are indicated by the corresponding-colored boxes (**b**, **d**, **f**).

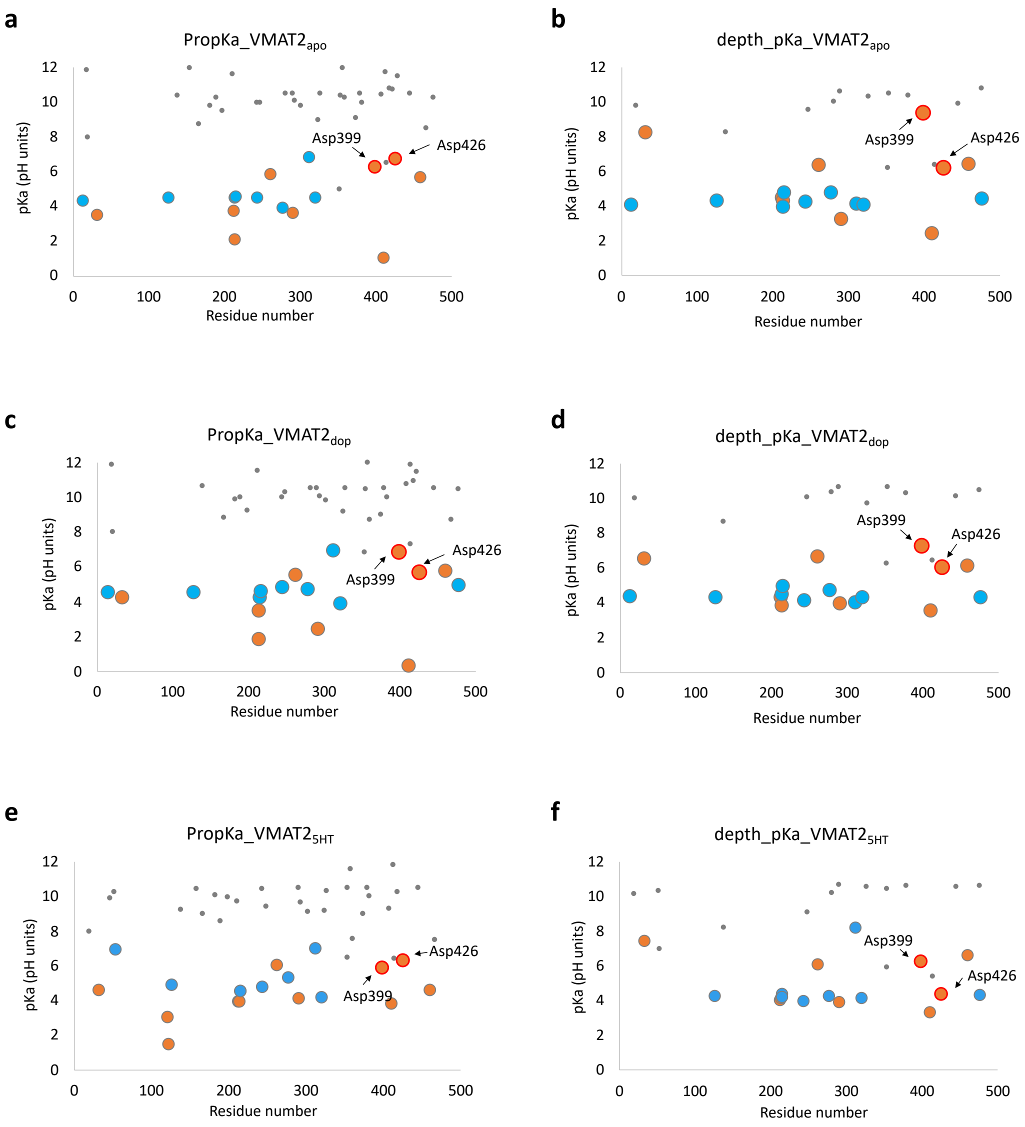

Supplementary Fig. 9 | pKa prediction. a-f, The pKa prediction server (**a, c, e**; https://www.ddl.unimi.it/vegaol/propka.htm, **b**, **d**, **e**; http://cospi.iiserpune.ac.in/depth) was used to predict the pKa of residues in VMAT2$_{apo}$ (**a**, **b**), VMAT2$_{dop}$ (**c**, **d**) and VMAT2$_{5HT}$ (**e**, **f**, PDB 8T6B). Asp is indicated by orange circles and Glu by blue circles.

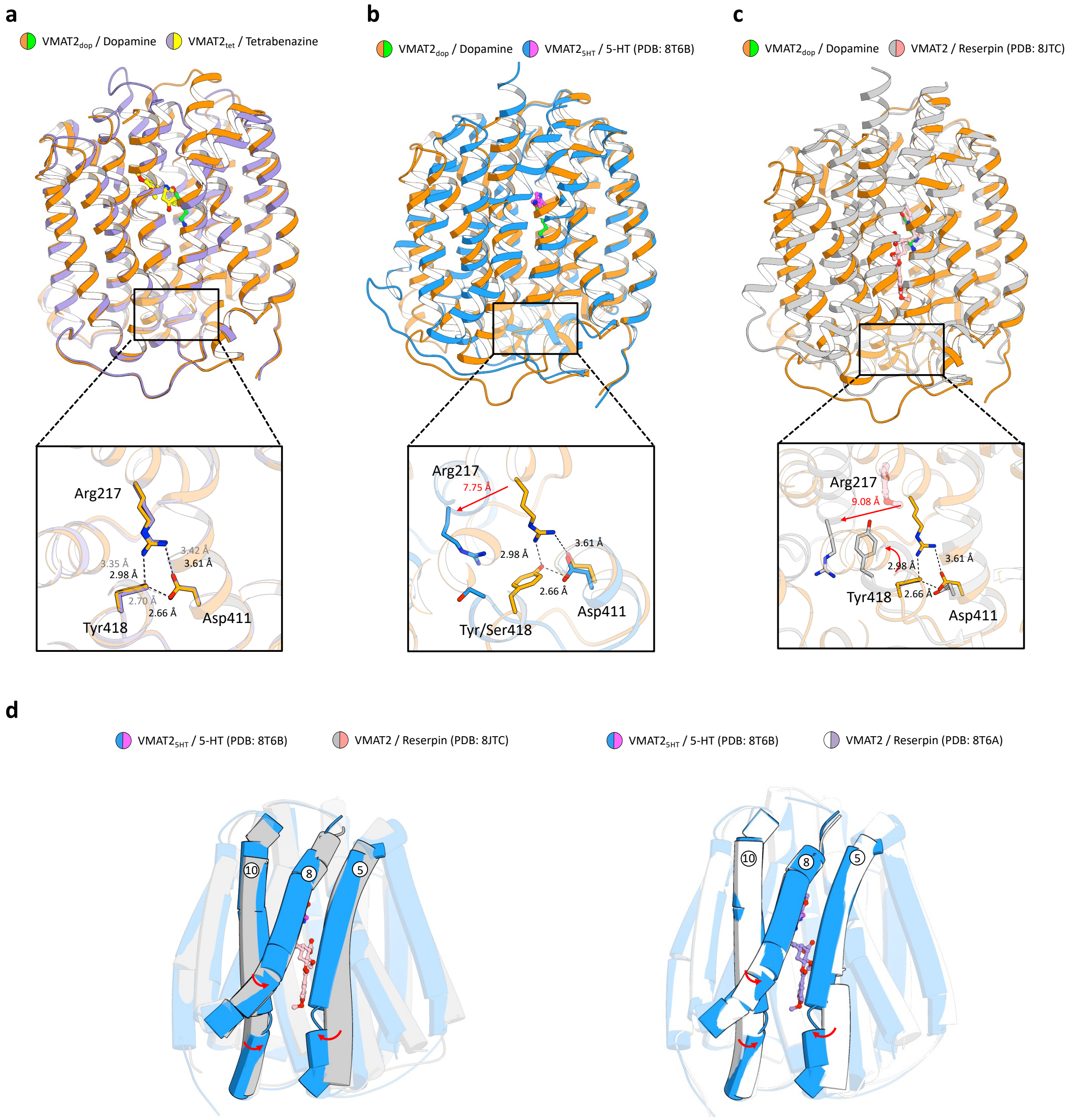

Supplementary Fig. 10 | Conformational changes in the intracellular region of VMAT2. a-c, Comparison of intracellular gate structures. VMAT2$_{dop}$ (orange) was superimposed with VMAT2$_{tet}$ (purple) (**a**), VMAT2$_{5HT}$ (blue, PDB 8T6B) (**b**) and reserpine-bound VMAT2 (gray, PDB 8T6A) (**c**), respectively. **d**, More "closed" structure of VMAT2$_{5HT}$. Superposition of VMAT2$_{5HT}$ and two different reserpine-bound VMAT2 structures (left panel; PDB 8JTC, right panel; PDB 8T6A). 5-HT and two reserpines are indicated by magenta, pink and purple sticks, respectively. The distances

between residues are shown as black dotted lines ($VMAT2_{dop}$) and gray dotted lines (**a**: $VMAT2_{tet}$, **b**: $VMAT2_{5\text{-}HT}$, **c**: reserpine-bound VMAT2).
