# Supplementary material for: Neurotransmitter recognition by human vesicular monoamine transporter 2
Source: Nat Commun. 2024 Sep 16;15:7661. doi: 10.1038/s41467-024-51960-z (PMC11405867; doi:10.1038/s41467-024-51960-z)
Supplement: Supplementary file 3 — Reporting Summary [file 41467_2024_51960_MOESM3_ESM.pdf]

nature portfolio

Corresponding author(s): Dohyun Im, So Iwata

Last updated by author(s): Jul 31, 2024

Reporting Summary

Nature Portfolio wishes to improve the reproducibility of the work that we publish. This form provides structure for consistency and transparency in reporting. For further information on Nature Portfolio policies, see our Editorial Policies and the Editorial Policy Checklist.

Statistics

For all statistical analyses, confirm that the following items are present in the figure legend, table legend, main text, or Methods section.

n/a	Confirmed	
☐	☒	The exact sample size (*n*) for each experimental group/condition, given as a discrete number and unit of measurement
☐	☒	A statement on whether measurements were taken from distinct samples or whether the same sample was measured repeatedly
☐	☒	The statistical test(s) used AND whether they are one- or two-sided<br>*Only common tests should be described solely by name; describe more complex techniques in the Methods section.*
☒	☐	A description of all covariates tested
☒	☐	A description of any assumptions or corrections, such as tests of normality and adjustment for multiple comparisons
☐	☒	A full description of the statistical parameters including central tendency (e.g. means) or other basic estimates (e.g. regression coefficient) AND variation (e.g. standard deviation) or associated estimates of uncertainty (e.g. confidence intervals)
☐	☒	For null hypothesis testing, the test statistic (e.g. *F*, *t*, *r*) with confidence intervals, effect sizes, degrees of freedom and *P* value noted<br>*Give P values as exact values whenever suitable.*
☒	☐	For Bayesian analysis, information on the choice of priors and Markov chain Monte Carlo settings
☒	☐	For hierarchical and complex designs, identification of the appropriate level for tests and full reporting of outcomes
☒	☐	Estimates of effect sizes (e.g. Cohen's *d*, Pearson's *r*), indicating how they were calculated

Our web collection on statistics for biologists contains articles on many of the points above.

Software and code

Policy information about availability of computer code

Data collection	Automated data collection was performed on the Titan Krios at 300 kV accelerating voltage in the Institute for Protein Research, Osaka University (Osaka, Japan).
Data analysis	Cryo-EM micrographs were recorded using a Gatan K3 detector in counting mode with a pixel size 0.88 Å using the SerialEM software (3.8). Structures were solved and refined using the Phenix software suite (1.18.X - 1.20.X) and Coot (0.8.0 and 0.9.5). Signaling assays were analyzed using Graphpad Prism 9. cryoSPARC (3.2.0 - 4.1.1), CueMol (2.2.3), and UCSF ChimeraX (1.5) were also used in this study.

For manuscripts utilizing custom algorithms or software that are central to the research but not yet described in published literature, software must be made available to editors and reviewers. We strongly encourage code deposition in a community repository (e.g. GitHub). See the Nature Portfolio guidelines for submitting code & software for further information.

Data

Policy information about availability of data

All manuscripts must include a data availability statement. This statement should provide the following information, where applicable:

- Accession codes, unique identifiers, or web links for publicly available datasets
- A description of any restrictions on data availability
- For clinical datasets or third party data, please ensure that the statement adheres to our policy

The atomic coordinates and the electron microscopy maps have been deposited in the Protein Data Bank (PDB) under accession number 7YFC and 7YFD and

Electron Microscopy Data Bank (EMDB) accession number EMD-33785 and EMD-33786 for he histamine-bound H4R-Gq complex and the imetit-bound H4R-Gq complex, respectively. Source data are provided with this paper.

Human research participants

Policy information about studies involving human research participants and Sex and Gender in Research.

Reporting on sex and gender	N/A
Population characteristics	N/A
Recruitment	N/A
Ethics oversight	N/A

Note that full information on the approval of the study protocol must also be provided in the manuscript.

Field-specific reporting

Please select the one below that is the best fit for your research. If you are not sure, read the appropriate sections before making your selection.

☒ Life sciences ☐ Behavioural & social sciences ☐ Ecological, evolutionary & environmental sciences

For a reference copy of the document with all sections, see nature.com/documents/nr-reporting-summary-flat.pdf

Life sciences study design

All studies must disclose on these points even when the disclosure is negative.

Sample size	Sample sizes were not predetermined by statistical methods. For cryo-EM data, sample size were determined by availability of microscope and images were collected until the resolution and 3D reconstruction converges. For all the functional assay, we use sample size at least of three independent experiments, commonly exploited by researches in this field.
Data exclusions	No data were excluded from the analysis.
Replication	Protein samples were purified from different purification batch and used for the cryo-EM study. The biochemical experiments in this study have been repeated by greater than or equal to three independent experiments, and those findings are reliably reproduced.
Randomization	Randomization was not relevant to this study, as the data were collected automatically and did not involve choosing.
Blinding	Blinding is not necessary or valid for the purposes of structural determination. For cryo-EM study, imaging data were collected automatically. For functional analysis, blinding was not necessary by reason of the quantitative nature of the experiment.

Reporting for specific materials, systems and methods

We require information from authors about some types of materials, experimental systems and methods used in many studies. Here, indicate whether each material, system or method listed is relevant to your study. If you are not sure if a list item applies to your research, read the appropriate section before selecting a response.

Materials & experimental systems

n/a	Involved in the study	
☐	☒	Antibodies
☐	☒	Eukaryotic cell lines
☒	☐	Palaeontology and archaeology
☐	☒	Animals and other organisms
☒	☐	Clinical data
☒	☐	Dual use research of concern

Methods

n/a	Involved in the study	
☒	☐	ChIP-seq
☐	☒	Flow cytometry
☒	☐	MRI-based neuroimaging

Antibodies

Antibodies used	anti-DYKDDDDK tag monoclonal antibody (FUJIFILM Wako Pure Chemical Corporation; Mouse; 014-22383; 1/400), Alexa Fluor™ 488 (Thermo Fisher Scientific; Goat; A-11029; 1/400)

Validation	The antibody used is commercially purchased and has been validated by the vendors. Validation data is available from the vendor's website.

Eukaryotic cell lines

Policy information about cell lines and Sex and Gender in Research

Cell line source(s)	Sf9 and HEK293 cells were purchased from Thermo Fisher Scientific.
Authentication	Non of cell lines were authenticated.
Mycoplasma contamination	Cell lines were tested and free from mycoplasma contamination.
Commonly misidentified lines (See ICLAC register)	None of commonly misidentified lines were used in this study.

Animals and other research organisms

Policy information about studies involving animals; ARRIVE guidelines recommended for reporting animal research, and Sex and Gender in Research

Laboratory animals	*For laboratory animals, report species, strain and age OR state that the study did not involve laboratory animals.*
Wild animals	*Provide details on animals observed in or captured in the field; report species and age where possible. Describe how animals were caught and transported and what happened to captive animals after the study (if killed, explain why and describe method; if released, say where and when) OR state that the study did not involve wild animals.*
Reporting on sex	*Indicate if findings apply to only one sex; describe whether sex was considered in study design, methods used for assigning sex. Provide data disaggregated for sex where this information has been collected in the source data as appropriate; provide overall numbers in this Reporting Summary. Please state if this information has not been collected. Report sex-based analyses where performed, justify reasons for lack of sex-based analysis.*
Field-collected samples	*For laboratory work with field-collected samples, describe all relevant parameters such as housing, maintenance, temperature, photoperiod and end-of-experiment protocol OR state that the study did not involve samples collected from the field.*
Ethics oversight	*Identify the organization(s) that approved or provided guidance on the study protocol, OR state that no ethical approval or guidance was required and explain why not.*

Note that full information on the approval of the study protocol must also be provided in the manuscript.

Flow Cytometry

Plots

Confirm that:

- ☐ The axis labels state the marker and fluorochrome used (e.g. CD4-FITC).
- ☐ The axis scales are clearly visible. Include numbers along axes only for bottom left plot of group (a 'group' is an analysis of identical markers).
- ☐ All plots are contour plots with outliers or pseudocolor plots.
- ☐ A numerical value for number of cells or percentage (with statistics) is provided.

Methodology

Sample preparation	Sample preparation listed in Methods.
Instrument	Guava EasyCyte Plus (Millipore)
Software	CytoSoft 5.3.1
Cell population abundance	Approximately 10,000 cellular events were collected and the total fluorescence intensity of positive expression cell population was calculated.
Gating strategy	Gating was determined by the Alexa Fluor™ 488 fluorescence intensity to differentiate positive cells and all other cells.

☒ Tick this box to confirm that a figure exemplifying the gating strategy is provided in the Supplementary Information.
